# Semantic Network Analysis Using Construction Accident Cases to Understand Workers’ Unsafe Acts

**DOI:** 10.3390/ijerph182312660

**Published:** 2021-12-01

**Authors:** Suhyun Kang, Sunyoung Cho, Sungmin Yun, Sangyong Kim

**Affiliations:** 1 School of Architecture, Yeungnam University, Gyeongsan-si 38541, Korea; yp043422@ynu.ac.kr (S.K.); csyung0929@ynu.ac.kr (S.C.); 2 Department of Civil Engineering, Yeungnam University, Gyeongsan-si 38541, Korea; smyun@yu.ac.kr

**Keywords:** construction accident case, construction workers, unsafe acts, semantic network analysis, HFACS, human error

## Abstract

Unsafe acts by workers are a direct cause of accidents in the labor-intensive construction industry. Previous studies have reviewed past accidents and analyzed their causes to understand the nature of the human error involved. However, these studies focused their investigations on only a small number of construction accidents, even though a large number of them have been collected from various countries. Consequently, this study developed a semantic network analysis (SNA) model that uses approximately 60,000 construction accident cases to understand the nature of the human error that affects safety in the construction industry. A modified human factor analysis and classification system (HFACS) framework was used to classify major human error factors—that is, the causes of the accidents in each of the accident summaries in the accident case data—and an SNA analysis was conducted on all of the classified data to analyze correlations between the major factors that lead to unsafe acts. The results show that an overwhelming number of accidents occurred due to unintended acts such as perceptual errors (PERs) and skill-based errors (SBEs). Moreover, this study visualized the relationships between factors that affected unsafe acts based on actual construction accident case data, allowing for an intuitive understanding of the major keywords for each of the factors that lead to accidents.

## 1. Introduction

The construction industry is one of the most hazardous industries in the world, and many studies have shown that unsafe behavior by people is a major cause of construction accidents [1,2,3,4,5]. These accidents can be attributed to unsafe conditions in the workplace, that is, physical factors such as when the workplace environment is inadequate, and unsafe acts by workers, that is, human factors such as inappropriate responses by workers to hazardous situations [6,7,8,9]. Unsafe acts are a direct cause of accidents, and they occur due to complex interactions between multiple factors including organizational factors, the workers’ physical condition, the work conditions, and the work environment [10,11,12]. The construction industry is a labor-intensive industry, and there are many tasks which workers perform directly, making it is necessary to understand human error, which is a fundamental cause of unsafe acts.

Human error generally means inappropriate or unwanted human decisions or actions, and it refers to mistakes, faults, slips, lapses, errors, etc. [13]. In order to understand such human error, studies have reviewed accident cases and analyzed their causes [10,14,15]. Based on these studies, a number of attempts have been made to develop models and methods for accident analysis in the construction sector, but these attempts have had several limitations.

One of the limitations is that previous studies have reviewed an inadequate number of construction accident case samples. Currently, construction accident case reports are becoming mandatory in many countries (United States, South Korea, Singapore, United Kingdom, Australia), and as a result, a large volume of construction accident information is being collected. However, these accident cases are excluded from studies as they consist of basic accident information and do not provide concrete evidence such as detailed on-site investigations and on-site expert testimony. Consequently, only a small number of cases which provide concrete evidence are selected for inclusion in studies. Another limitation is that studies have focused on surveys of workers and managers, most having been conducted using self-reporting questionnaires to measure the workers’ behavior. These drawbacks limit the sampling targets and can lead to various forms of bias.

This study aimed to use a large number of construction accident cases to analyze the causes of unsafe behavior by workers due to the complex interactions between major factors. The goal of this study was to use actual construction accident case data to develop a semantic network analysis (SNA) model and examine the core factors which lead to unsafe acts by workers. By doing so, it will be possible to understand the correlations between human factors that affect safety. Moreover, the data visualized through network graphs should be able to provide intuitive information that is different from that provided using previous statistical methods.

## 2. Literature Review

In the field of construction safety, studies are being conducted to understand the causes of unsafe acts by workers using various accident analysis models. Existing studies have collected survey data on accidents from workers and experts and used statistical analysis methods to understand their causes. Harvey et al. (2018) updated and reviewed the reliability of the construction accident causation (CONCA) model by conducting interviews with 32 construction safety managers and consultants to understand the causes of construction accidents. Zhang et al. (2019) created a construction accident cause system (CACS) model and used systemic accident, statistical analysis, and case study methods on 571 construction accidents to identify the causes of serious accidents. Wang et al. (2016) performed a survey on 297 workers to discover the fundamental causes of unsafe acts, using structural equation modeling (SEM) to identify the important factors and paths affecting the workers’ range of safety risk tolerances. Park et al. (2020) used logistic regression methods to examine correlations between unsafe acts and unsafe conditions and their significance in influencing construction accidents. However, the study had the drawback of only considering one-to-one combinations of these phenomena and being unable to examine anything beyond one-dimensional relationships, with the correlations between various causes remaining unclear.

These studies were able to understand the causes of construction accidents based on the opinions of workers and experts, but insufficient effort was made to find causes within a large volume of actual accident cases. Moreover, the normal statistical analysis methods used for examining construction accident cases tend to result in the loss of information (due to simplification) regarding accident types and cause analyses. Statistical methods limit the analysis of accident case data by simplifying rich information—such as linguistic meanings and the various relationships between factors—contained in accident reports that are described in a written (sentence) format.

Various accident analysis models have been used to identify the causes of industrial accidents, including the domino theory (Heinrich 1969), the Ferrel theory (Heinrich 1980), the ‘Swiss cheese’ model of human error (Reason 1990), and the human factor analysis and classification system (HFACS) (Shappell and Wiegmann 1997, 2000). Of these, the HFACS was developed as a tool for analyzing human error in aviation accidents and has been widely cited in various fields (railways, mining, maritime shipping, healthcare, etc.), including construction [15,16,17,18,19]. Wong et al. (2016) adopted and modified the HFACS to classify the fundamental causes of 52 fatal fall-from-height (FFH) accidents which included on-site expert testimony and supplemental reports. Ye et al. (2018) emphasized the need for a systemic approach to examining human error in order to improve construction safety, and they developed an improved I-HFACS framework and used it to classify 150 accident cases and perform frequency analysis. Xia et al. (2018) also modified the HFACS framework to create the BN-HFACS model—that is, a combination of HFACS and a Bayesian network (BN)—to predict the safety performance of construction projects. Baldissone et al. (2019) classified data based on the HFACS classification system to develop an accident precursor management system. In addition to these studies, many other construction safety studies have used the HFACS to analyze and prevent accidents. In this way, previous studies have shown the HFACS to be useful for providing systematic analysis of construction accident causes. Consequently, this study aimed to use a modified HFACS framework to classify the major human error factors that cause accidents discussed in the accident summaries of construction accident case data.

## 3. Semantic Network Analysis (SNA)

SNA is an analysis method for finding significant structural relationships between words in text that consists of messages [20]. SNA calculates the frequency of words in sentences to analyze the patterns with which words create structure and meaning. It is useful in identifying the relationships between words through text analysis and understanding the relationships between core concepts within the overall context. It also has the advantage of allowing the results to be presented in a manner that is intuitive and easy to understand by using various visualization techniques.

In SNA, certain concepts are depicted as nodes, and the relationships between these concepts are depicted as links. By depicting the concepts as nodes and links, the role of each concept and the correlations between them can be established. The relationships between words are shown through the phenomenon of co-occurrence in which words appear together in a certain text unit [21]. Moreover, SNA objectively determines the relationships between causes by detecting the patterns or behaviors of major causes without bias, based on actual text-format data rather than theoretical background information [22].

However, text is difficult to analyze by itself as it is unstructured data. Consequently, a mining process for extracting information is needed. Text mining is a process that converts the unstructured data of text into a structured format to find useful information [23]. A computer uses natural language processing (NLP) technology to convert the unstructured data into a format that it can understand, after which it processes, extracts, and analyzes the information. At this point, SNA is used in the text extraction and analysis stage. The aforementioned features make SNA the most suitable method for analyzing the relationships between human error factors that lead to unsafe acts based on large amounts of text-format construction accident case data in this study.

## 4. Classification of Modified HFACS and Network Modeling

### 4.1. Research Methodology

**Figure 1:**
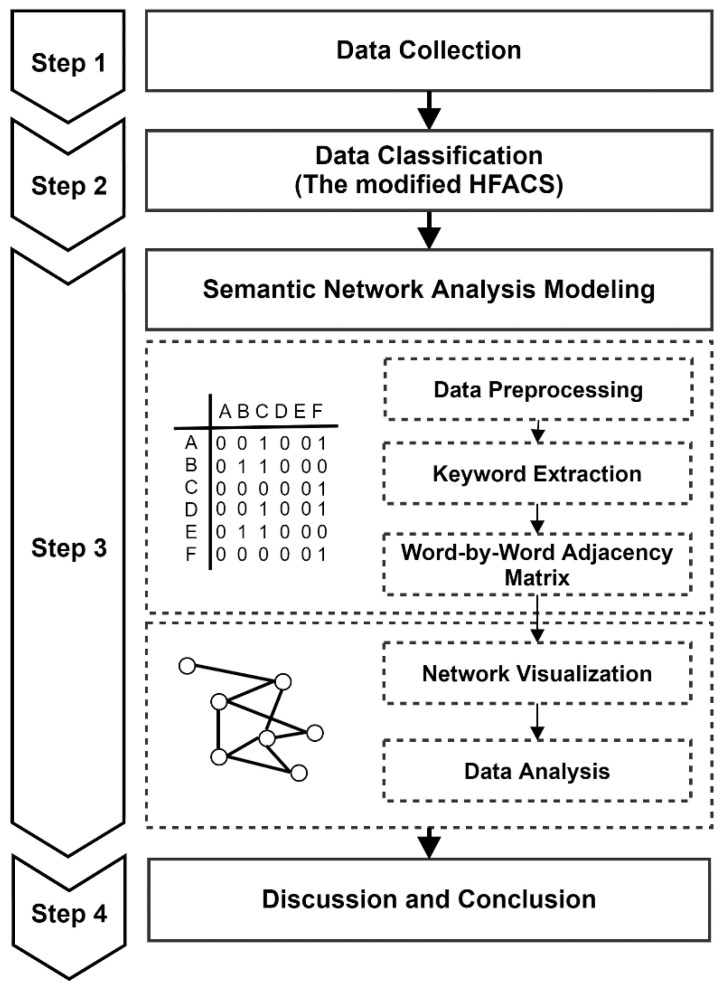
Research process.

This study aimed to use SNA to perform data analysis based on the simple hypothesis that there is a higher correlation between different causes if they appear together in a single accident case. Figure 1 shows the process of developing the SNA model, which was the goal of this study.

In the data collection stage, construction accident cases from 2014 to 2018 collated by the Korea Occupational Safety and Health Agency (KOSHA) were collected, and approximately 60,000 suitable data were finally selected. The collected data were text data in which the accident summaries were comprehensively recorded in sentence form, including the various accident causes. To analyze the accident causes within such an unstructured dataset, significant manual classification was required. Consequently, during the data classification stage, the collected data were manually classified by 15 specialized human resources, based on the modified human factor analysis and classification system (HFACS) framework consisting of 4 levels—that is, unsafe behavior, preconditions of unsafe acts, unsafe supervision, and organizational influences—and 17 sub-factors. In the SNA modeling stage, two big data analysis techniques—that is, text mining and semantic network analysis—were used to analyze large volumes of text data. In the text mining stage, data preprocessing was performed using Python 3.9.0, and morphemes were analyzed using the KoNLPy library. Subsequently, words that were targets for detailed analysis were extracted. Meaningless words and noise that were extracted during the morpheme analysis process were removed to identify keywords among the analyzed data. After preprocessing was completed, top-level factors appearing frequently were selected from among the extracted keywords, and the Numpy and Pandas libraries were used to examine the frequency at which these words appeared simultaneously and to arrange connection relationships in the form of an adjacency matrix. In the SNA stage, the extracted adjacency matrix was visualized using Gephi 0.9.2—an open-source network analysis tool—to create a network showing the connection relationships between keywords, with the major content then being analyzed.

### 4.2. Finding the Property Information of Construction Accident Data

This study collected a total of 89,355 data (88,007 accident cases, 1348 fatal cases) to analyze the human error factor in construction accident cases. Ultimately, 60,183 data were selected for analysis after excluding data that had been duplicated or could not be checked—that is, the accident summary was very short, or information was missing. The collected accident case data include a large volume of unnecessary information; therefore, the required property information was found based on the general construction information (project scale, region), incident information (date of accident, time of accident, accident summary, number of dead and injured people), worker information (length of service, type of employment, age), and hazard (disability level, number of working days lost).

**Figure 2:**
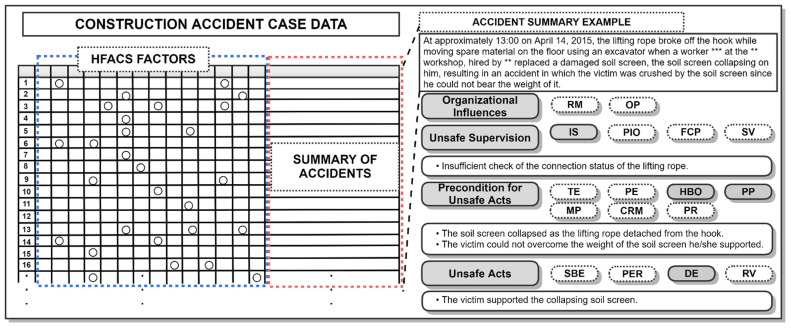
Example of the HFACS factor classification method.

The main type of information used in this study was accident summaries. In the accident summaries, it was possible to see the major factors and causes of unsafe acts based on the accident circumstances. The accident summary describes the type of accident—such as a fall, collapse, pinching, falling—including where, when, and in what place the accident occurred, during what process, with what type of machine/equipment, and doing what type of work. In particular, it includes any unsafe conditions regarding machinery, equipment, structures, and the working environment at the time of the accident and any specific human factors relating to the unsafe behavior of the injured or fellow workers. The collected accident case data contain sufficient information to analyze the causes of HFACS-based construction accidents despite the various levels of detail used to describe each accident. As such, the cases caused by specific human error factors were classified based on the modified HFACS framework (which included 17 factors). At this stage, the accident cases were arranged as rows, and the human error factors (17 factors) were arranged as columns. When a certain factor contributed to an accident in an accident case, the corresponding text content was entered in that factor’s cell. Figure 2 shows an example of the HFACS factor classification method. The accident summary shown indicates that the safety management supervisor failed to check the (insufficient) status of rope connections, which is clearly their responsibility. This caused an unexpected situation in which the lifting rope broke off a hook and a soil screen collapsed, resulting in an accident which injured a worker, even though the victim was aware of the danger of collapse. As a result of the classification, four sub-factors were identified as the cause of the accident—that is, inadequate supervision, hazard by others, physical problem, and decision error—suggesting that the accident occurred due to the combined influence of each factor. After classification was completed, SNA was performed on the accident case data, the data being used to find correlations in the unsafe acts for each of the HFACS factors.

### 4.3. Classification of Causes Based on the Modified HFACS for Unsafe Acts

When the HFACS was first developed, it was classified into 20 sub-items within 4 major categories: that is, (1) unsafe acts, (2) preconditions for unsafe acts, (3) unsafe supervision, and (4) organizational influences [24,25]. When the HFACS is used, it has the advantage of being able to distinguish between latent failures and active (human) failures at each level. Latent failures include factors such as inadequate organizational management practices, inadequate or missing resources, supervisor violations, inadequate equipment design, and inadequate personnel training and procedures. Conversely, active (human) failures include unsafe acts that occur close to the moment when an accident occurs.

**Figure 3:**
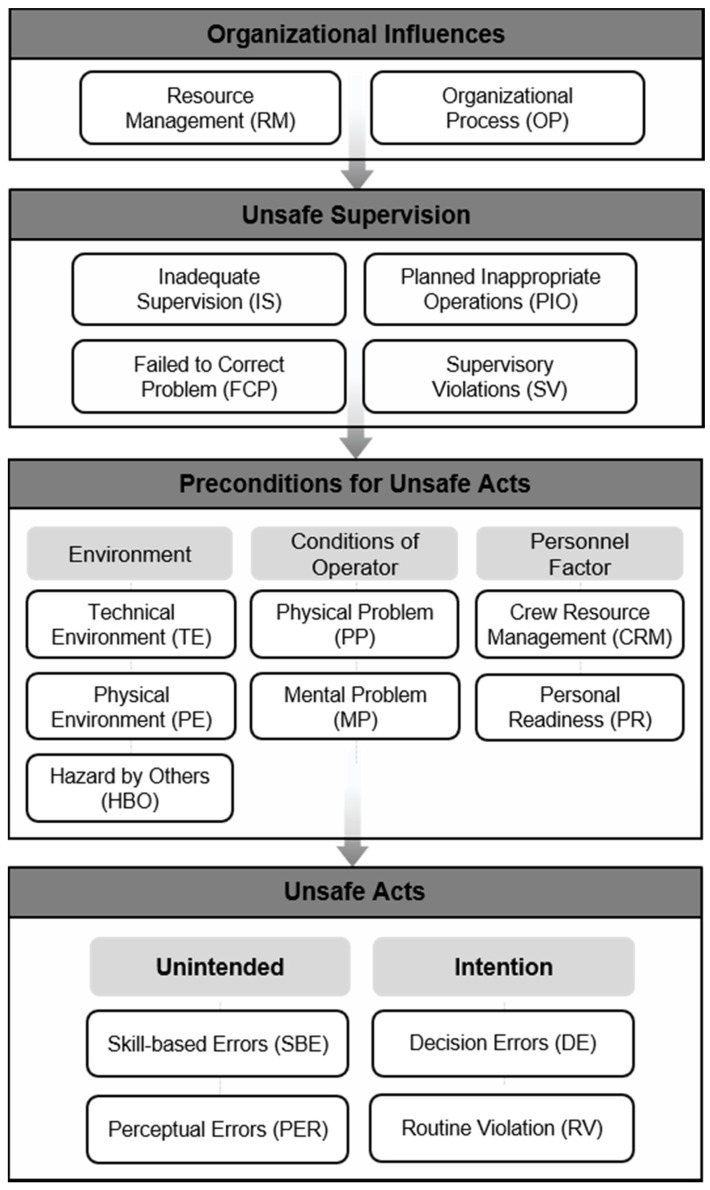
The modified HFACS used in this study.

This study modified the classification system based on the HFACS framework used by Wong, L. et al. (2016). In their version of the HFACS framework, there were a total of 20 sub-items, but this study modified their version by excluding items that could not be known from the accident case data, with 17 sub-items being chosen (Figure 3). The meanings of each sub-item are shown in Table 1.

The two excluded sub-items were ‘organizational climate’ and ‘exceptional violation.’ ‘Organizational climate’ in ‘(4) organizational influences’ was excluded because it is difficult to understand the inappropriate organizational safety structures and politics or cultural factors within an organization. In addition, ‘exceptional violation’ in ‘(1) unsafe acts’—which refers to exceptional violations of established rules and procedures—was excluded for the same reason. Moreover, the three sub-factors (‘adverse mental states (ADM),’ ’adverse physiological states (ADP),’ ’physical/mental limitations (PML)’) in the subcategory ‘conditions of operator’ under ‘(2) preconditions for unsafe acts’ were changed to the more inclusive terms ‘physical problem (PP)’ and ‘mental problem (MP)’ as it is difficult to understand the workers’ individual state of health or specific psychological state.

The biggest change in this study’s modified HFACS framework was ‘(1) unsafe acts.’ Most of the studies that have used the HFACS framework divided ‘(1) unsafe acts’ into ‘errors’ and ‘violations,’ as was conducted in the existing classification system [26]. According to Fogarty and Shaw (2010), the frequency of ‘violations’ was generally lower than the frequency of ‘errors,’ and in this study’s data too, the frequency of ‘violations’ was very low; consequently, in this study, ‘(1) unsafe acts’ were primarily classified as ‘errors.’ In general, the classification of human error varies based on one’s concept of ‘error’ and perspective, with there being no universally accepted classification. James Reason, who pioneered formal research on human error, wrote that the role of ‘intention’ must be considered first when considering ‘errors’ [27]. As Reason asserted, research on ‘intention’ is important for understanding human error. Therefore, this study classified the sub-items under ‘(1) unsafe acts’ by focusing on whether the worker’s unsafe act was intended or unintended. The two sub-factors for unintended unsafe acts were ‘skill-based errors (SBEs)’ and ‘perceptual errors (PERs),’ and the sub-factors for intended unsafe acts were ‘decision errors (DEs)’ and ‘routine violations (RVs)’ (Figure 3).

## 5. Semantic Network Analysis Results

### 5.1. Overall Data Social Network Analysis Results

**Figure 4:**
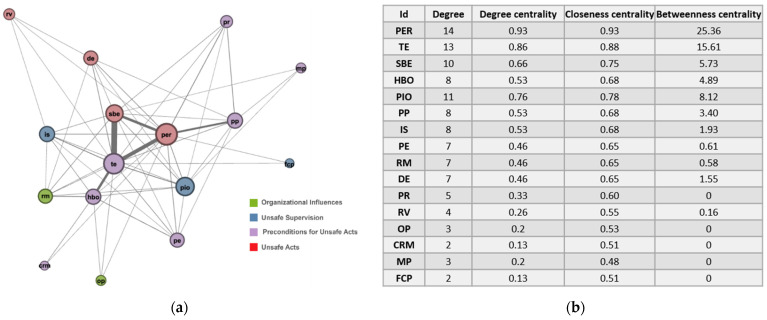
Holistic social network analysis result: (**a**) network analysis diagram; (**b**) network centrality analysis table.

Figure 4a shows the overall network diagram, and Figure 4b shows the degree, degree centrality, closeness centrality, and betweenness centrality values for each factor in the SNA results. A total of 16 nodes appeared in the approximately 60,000 data—except for ‘supervisory violations (SVs),’ which had less than 50 instances—and 56 links were formed between the nodes. It is thought that there were so few SVs because managers excluded their own mistakes when reporting accidents in most cases.

As a degree centrality value increases, the size of the node in Figure 4a becomes larger, representing the frequency at which the factor appears in the overall accident case data. Degree is the number of directly connected nodes, its value being visualized in Figure 4a. Among the four factors in ‘(1) unsafe acts’ in the network graph, the node with the largest node size and highest degree centrality was ‘PER,’ meaning that most of the accidents among the accident cases occurred due to misunderstanding of objects and threats or a lack of cognitive ability (mistakes in seeing or hearing). The ‘PER’ factor also had high closeness centrality and betweenness centrality in addition to high degree centrality. Closeness centrality refers to how closely a node’s degree of closeness is connected to all other nodes in the network [28]. In other words, if closeness centrality was high, the node could be said to be the closest to other nodes in the network, meaning that the node could affect or be affected by other nodes the most quickly. Betweenness centrality is a method of measuring whether a node performs the role of a bridge (intermediary) with other nodes when building the network [29]. The more often a node appears on the shortest path between other nodes, the higher the node’s betweenness centrality—that is, a node with high betweenness centrality has control over the flow of information, this node having a significant effect on the network’s overall connections and flow. Of the four factors under ‘(1) unsafe acts’ in the network, the factor with the second-highest centrality was ‘SBE,’ the two factors ‘PER’ and ‘SBE’ being unintended unsafe acts in the category of ‘(1) unsafe acts.’ From this, it can be surmised that a larger number of accidents were caused by unintended unsafe acts in comparison to intended unsafe acts.

### 5.2. The Key Factors Affecting Unsafe Acts

To understand the strength of the relationships between other factors that affect the factors in ‘(1) unsafe acts,’ the ‘direction’ of the relationships was set under the assumption that unsafe acts were affected in the order of ‘organizational influences’ → ‘unsafe supervision’ → ‘preconditions for unsafe acts’ → ‘unsafe acts’. A directed network contains relationships that have directions wherein a starting point and an end point exist. In an undirected network, the relationships between two nodes are the same for each node. In this study’s data, there were four factors in the category of unsafe acts, and the factors which lead to unsafe acts were divided based on whether they were intended or not. The unintended unsafe acts include skill-based errors (SBEs) and perceptual errors (PERs), and the intended unsafe acts include decision errors (DEs) and routine violations (RVs). The SNA analysis was conducted based on these categories, the results being as follows.

**Figure 5:**
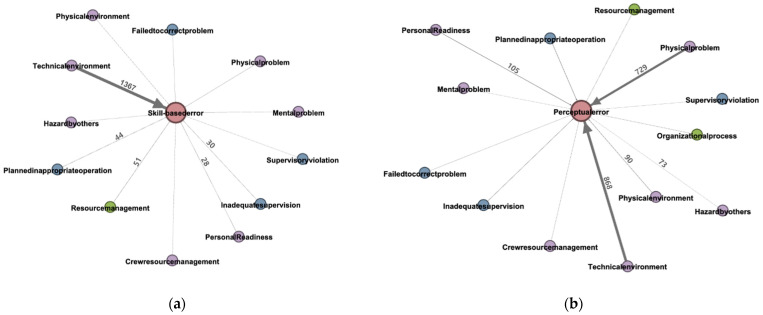

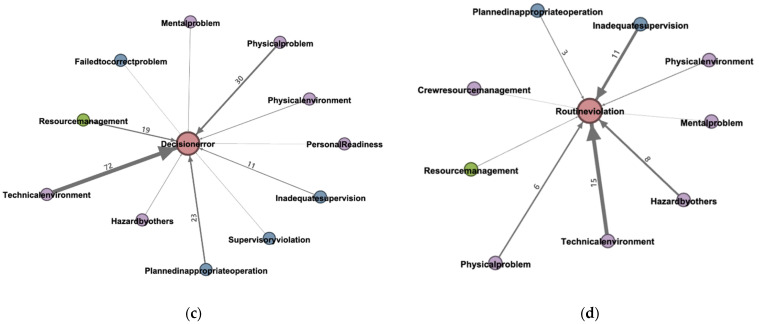
Network diagrams of unsafe act factor analysis results: (**a**) skill-based errors (SBEs) network diagram; (**b**) perceptual errors (PERs) network diagram; (**c**) decision errors (DEs) network diagram; (**d**) routine violations (RVs) network diagram.

‘Skill-based errors (SBEs)’ are unintended unsafe acts. They are errors which can occur as a worker performs an action in a skilled state, and they can be divided into slips and lapses in short-term memory. Typical examples include getting one’s gloves caught in a drill during drilling or forgetting to tighten a screw. Figure 5a shows the weights of the factors having the most influence on ‘SBEs’ when the directionality of the relationships between nodes is set. The factors having the most influence on ‘SBEs’ are ‘TE (1367),’ ‘RM (51),’ and ‘PIO (44),’ in that order.

The ‘perceptual errors (PERs)’ in Figure 5b are also unintended unsafe acts, and they refer to poor recognition of objects, hazards, or situations, including impairments to sight, hearing, cognition, and attention. Typical examples include being unable to see a chainsaw blade due to the glare of the sun or being unaware of a working crane behind oneself due to ambient noise. In Figure 5b, the factors having the most influence on ‘PERs’ are ‘TE (868),’ ‘PP (729),’ and ‘PR (105),’ in that order.

In Figure 5c, ‘decision errors (DEs)’ are intended unsafe acts, and they mean that the selected plan is unsuitable or improper for achieving the desired results. These errors do not occur because the technology for performing tasks is inadequate. Rather, they include decisions that exceed capacity, improper responses to emergency situations, and inadequate procedural decisions. Typical examples include climbing on scaffolding outside of the designated path and falling, or falling after a pipe gives way while working above an exhaust duct. In Figure 5c, the factors having the most influence on ‘DEs’ are ‘TE (72),’ ‘PP (30),’ and ‘PIO (23),’ in that order.

In Figure 5d, ‘routine violations (RVs)’ are intended unsafe acts, and they mean that the specified rules and procedures have been intentionally ignored. Typical examples include working without wearing a hardhat or having a brick fall and strike the head when the hardhat has fallen off because of an unfastened chinstrap. In Figure 5d, the factors having the most influence on ‘RVs’ are ‘TE (15),’ ‘IS (11),’ and ‘HBO (8),’ in that order.

Next, the major factors that have an effect on unsafe acts at each of the major category levels in the HFACS are described based on the analysis results shown in Figure 5.

(1) Organizational Influences: The factor having the most influence on ‘(1) unsafe acts’ at the level of ‘(4) organizational influences’ is ‘resource management (RM).’ ‘RM’ refers to problems in the acquisition, distribution, and management of the organization’s resources. Typical examples include inadequate resources or the provision of faulty or worn-out equipment.
(2) Unsafe Supervision: The factors that influence ‘(1) unsafe acts’ at the level of ‘(3) unsafe supervision’ are ‘planned inappropriate operation (PIO)’ and ‘inadequate supervision (IS)’. ‘PIO’ refers to causing unnecessary hazards for on-site workers due to defective task planning. Examples include excessive workload orders and inappropriate personnel arrangements. ‘IS’ refers to inappropriate supervision that causes workers to be unable to recognize and control hazards. A typical example is lax safety supervision.
(3) Preconditions for Unsafe Acts: The factors that influence ‘(1) unsafe acts’ at the level of ‘(2) preconditions for unsafe acts’ are ‘technical environment (TE),’ ‘physical problem (PP),’ ‘hazard by others (HBO),’ and ‘personal readiness (PR).’ ‘TE’ refers to work environment factors (workspace, design factors) that affect a worker’s individual job. Typical examples include getting caught in a machine due to limited space or machine failure. ‘Physical problem (PP)’ refers to workers’ bodily limitations (reduced stamina, alcohol, drug usage, etc.). ‘Hazard by others (HBO)’ refers to hazardous situations created not by the victim of the accident but by others. Examples include faults in the safety status of a structure or bricks falling from the second floor. ‘Personal readiness (PR)’ refers to workers’ physical stamina and stress (time pressure), as well as insufficient rest.

### 5.3. Semantic Network Analysis Results of Unsafe Acts

**Figure 6:**
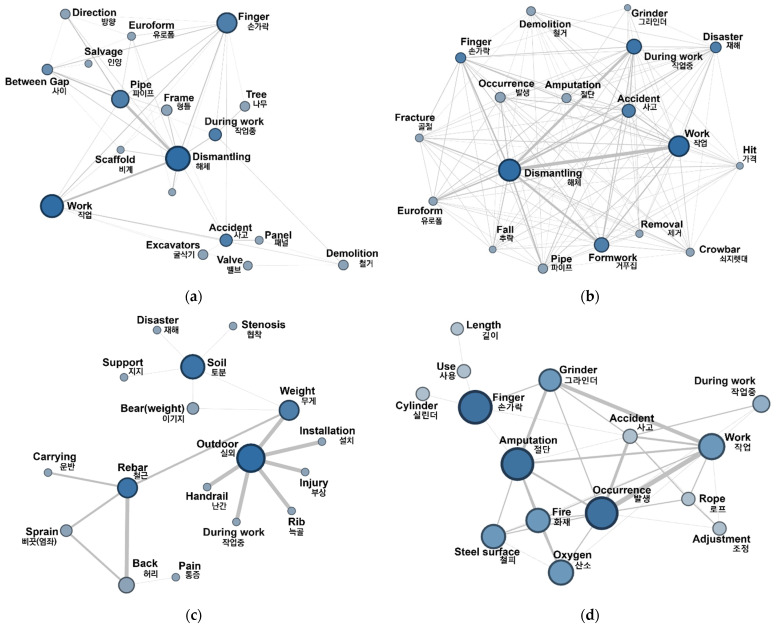
Semantic network analysis diagram of key factors affecting unsafe acts factor: (**a**) TE-PER semantic network diagram; (**b**) TE-SBE semantic network diagram; (**c**) PP-PER semantic network diagram; (**d**) PIO-SBE semantic network diagram.

Figure 6 and Table 2 show the results of performing an SNA on accident cases containing the three factors (TE, PP, PIO) that have the greatest influence on unsafe acts. Table 2 shows the result of centrality analysis of the SNA diagram. The initial SNA diagram exhibited too many links and nodes; therefore, it was reduced to an analyzable level by adjusting the connection strength between nodes.

(1) Technical environment (TE)-PER/SBE: The combinations ‘TE’ and ‘SBE’ (weight: 1367) and ‘TE’ and ‘PER’ (weight: 868) had the highest weight values among the mutual relationships between the HFACS factors. The analysis results for these two combinations were similar. Figure 6a,b show network graphs that display their major keywords and the correlations between them. It can be seen that most of the extracted keywords that both factors had in common were from accidents that occurred during demolition and dismantling work (18.63%) and installation work (12.46%). In particular, the values of nodes such as ‘dismantling,’ ‘during work,’ ‘(scaffold) pipe,’ ‘formwork,’ and ‘finger’ were large, the connections between these nodes being prominent. This phenomenon shows that the degree of hazard caused by work environment factors (TE) (upper floor work, narrow work environments, lack of work platforms and fall prevention nets, hazards from falling material, etc.) was high in demolition and dismantling work in comparison to other types of work. Such hazardous work environment factors during dismantling work may combine with the ‘PER’ factor (for example, being unable to see material falling from above) and the ‘SBE’ factor (such as mistakes in the sequence of dismantling tasks due to unskilled labor) to cause fatal accidents.
(2) Physical problem (PP)-PER: The combination ‘PP’ and ‘PER’ (weight: 729) had the third highest weight among the mutual relationships between the HFACS factors. This combination’s network graph is shown in Figure 6c, and it can be seen that node values for ‘outdoor,’ ‘weight,’ ‘rebar,’ ‘carrying,’ ‘back,’ and ‘strain’ were large and the connections between them prominent. This shows that inadequate physical capacity (PP) for performing certain work often occurs when handling heavy materials (such as rebar) during outdoor work that is affected by weather (such as heat waves). Moreover, it can be seen that when physical capacity reductions occur, there is an increased possibility of misunderstanding objects, threats, and situations as well as visual, auditory, cognitive, and attention deficits (PERs) at the same time, this being linked to not only light injuries (such as back strain) but also large secondary accidents (such as slipping due to momentary dizziness). However, there are many cases in which accidents happen because workers overestimate their own physical capacities and continue to work. A typical example is a worker straining physically but thinking that they are fine. The worker continues to work before suddenly losing strength and collapsing.
(3) Planned inappropriate operation (PIO)-SBE: The combination ‘PIO’ and ‘SBE’ (weight: 105) had the fourth highest weight among the mutual relationships between the HFACS factors. This combination’s network graph is shown in Figure 6d, and it can be seen that node values for ‘grinder,’ ‘finger,’ ‘amputation,’ ‘oxygen,’ and ‘fire’ are large and the connections between them strong. In work plan faults (PIO), there are many cases where accidents occur because unskilled workers are provided, or workers perform unexpected work due to a manager’s work adjustments. These situations occur at small- to mid-sized work sites. Typical examples of accidents include frequent finger amputation accidents when using grinders and fire accidents when using oxygen cutting equipment.

## 6. Conclusions

This study performed a network analysis using approximately 60,000 construction accident case data to identify the human error affecting unsafe acts by construction workers. Network analysis provided an approach which could compensate for the problems of previous studies—such as the limited number of construction accident case samples and the reliance on surveys and statistical analysis. This study used a modified HFACS framework to classify the major human error factors which were the causes of accidents for each accident case in the accident case data. An analysis was performed primarily on ‘(1) unsafe acts,’ one of the modified HFACS framework’s four levels—that is, unsafe acts, preconditions for unsafe acts, unsafe supervision, and organizational influences—and the analysis focused on whether the workers’ unsafe acts were intended or unintended.

The analysis results show that ‘PERs’ and ‘SBEs’ were the nodes with the largest node size and highest degree among the four factors belonging to ‘(1) unsafe acts’ in the network. These two factors were classified as unintended unsafe acts from among ‘(1) unsafe acts.’ It can be seen that an overwhelming number of accidents were caused by unintended unsafe acts as compared to intended unsafe acts. An SNA was performed on ‘TE,’ ‘PP,’ and ‘PIO’—which were the factors having the most influence on unsafe acts. As a result, the keywords that were the causes of the accident case factors were identified. This study was able to identify the cause materials and accident types associated with accident cases and present an analysis of the results as well as an approach for accident countermeasures.

It is expected that text-based analysis using a large volume of accident cases (accident report documents) will play an important academic and practical role in establishing new safety management systems. Previous studies have attempted to analyze all the causes of accidents within each focus, and no methodology has been provided to quantitatively and qualitatively analyze the interrelationships between the causes that lead to accidents. However, the SNA graph pattern of this study can identify the relationship between human factors that affect workers’ unsafe behavior within actual accident cases. This intuitively grasps the main keywords that cause accidents for each factor and provides a scenario for the process in which accidents occur. Furthermore, this can be used to identify possible accident occurrence scenarios for each major factor in the future when establishing accident prevention measures.

However, this study took a significant amount of time and resources to classify the causes of the accidents considering various factors and has a disadvantage in that it is difficult to check the consistency of the data. Consequently, based on the results of this study, a text mining-based automatic classification study of accident causes would be needed in the future, in order to improve the consistency, productivity, and efficiency of the process. One such study could standardize the various words that are used in the construction industry to automate data classification. Such a study would alleviate the classification and analysis difficulties that exist as a wide variety of different words are used in construction accident cases based on the accident reporter and the construction site (even though the words may have the same meaning). Moreover, it will be necessary to conduct studies on preventing unintended unsafe acts. This study’s results show that unintended unsafe acts occur often, making it much more difficult to establish preventive measures for unintended acts than intended acts. Consequently, it has been determined that there is a need for various studies on methodologies for preventing unintended unsafe acts.

## Figures and Tables

**Table 1:** The modified HFACS used in this study.

Classification	Factors	Descriptions
① Unsafe acts	Decision error (DE)	Actions and plans intentionally chosen by operators are inappropriate and lead to unsafe situations.
	Skill-based error (SBE)	Unintentional errors that can be reduced through learning. The actions are related to a routine task or procedure.
	Perceptual error (PER)	Misperception of an object, equipment, environment, threat, or situation; visual, auditory, proprioceptive, or vestibular illusions; cognitive or attention failures.
	Routine violation (RV)	Intentionally ignoring established rules and procedures.
② Precondition of unsafe acts	Physical environment (PE)	The environmental factor conditions that affect the actions of individuals.
	Technical environment (TE)	The workspace that affects the actions of individuals.
	Hazard by others (HBO)	Risks that, unknown to the victim, were caused by another party.
	Mental problem (MP)	Lack of mental capabilities to cope with a situation when performing certain tasks.
	Physical problem (PP)	Lack of physical capabilities to cope with a situation when performing certain tasks.
	Crew resource management (CRM)	Factors that include communication, coordination, planning, and teamwork issues.
	Personal readiness (PR)	Preparatory actions or behavior by an individual in order to perform safe work, such as abstaining from drinking or taking sufficient rest before work.
③ Unsafe supervision	Inadequate supervision (IS)	Inappropriate supervision that fails to control the risk of workers.
	Planned inappropriate operation (PIO)	Inappropriate work plans that pose unnecessary risks to workers.
	Failed to correct problem (FCP)	Failure to correct this problem even though defects in personal, equipment, training, or related safety issues are known to the supervisor.
	Supervisory violation (SV)	The intentional violation of existing regulations/rules by the supervisor.
④ Organizational influence	Resource management (RM)	Matters related to decision making with regard to the budget and resource distribution at the organizational level.
	Organizational process (OP)	Official processes at the organizational level, including safety management, safety education and training, operation speed, and work schedule.

**Table 2:** Centrality analysis result of semantic network analysis on accident cases.

	TE-PER				TE-SBE				PP-PER				PIO-SBE		
	DC	BC	CC		DC	BC	CC		DC	BC	CC		DC	BC	CC
Dismantling	0.10	0.06	0.21	Dismantling	0.29	0.09	0.39	Outdoor	0.11	0.02	0.13	Finger	0.16	0.18	0.31
Work	0.10	0.09	0.22	Work	0.27	0.09	0.40	Rebar	0.08	0.04	0.13	Occurrence	0.16	0.04	0.26
Finger	0.08	0.06	0.21	Working	0.19	0.03	0.36	Weight	0.07	0.05	0.15	Amputation	0.16	0.09	0.30
Pipe	0.07	0.03	0.19	Formwork	0.19	0.04	0.35	Soil	0.06	0.01	0.11	Work	0.12	0.06	0.25
During work	0.05	0.03	0.18	Accident	0.17	0.03	0.35	Bear	0.03	-	0.11	Oxygen	0.12	0.13	0.25
Frame	0.04	0.03	0.18	Disaster	0.13	0.02	0.34	Back	0.03	0.01	0.09	Steel surface	0.11	0.01	0.24
Tree	0.04	0.02	0.16	Finger	0.12	0.02	0.33	Handrail	0.02	-	0.09	Fire	0.11	0.07	0.24
Direction	0.04	-	0.17	Occurrence	0.12	0.02	0.33	Rib	0.02	-	0.09	Grinder	0.10	0.05	0.28
Excavators	0.04	0.05	0.17	Pipe	0.11	0.01	0.32	Injury	0.02	-	0.09	During work	0.08	0.01	0.22
Valve	0.03	0.02	0.17	Amputation	0.10	0.02	0.32	Sprain	0.02	-	0.09	Accident	0.06	0.05	0.28

DC: degree centrality; BC: betweenness centrality; CC: closeness centrality.

## Data Availability

Not applicable.
